# Impact of epidural labor analgesia using sufentanil combined with low-concentration ropivacaine on maternal and neonatal outcomes: a retrospective cohort study

**DOI:** 10.1186/s12871-021-01450-2

**Published:** 2021-09-22

**Authors:** Le Zhang, Chengjie Xu, Yue Li

**Affiliations:** Department of Anesthesiology, Obstetrics and Gynecology Hospital of Fudan University, 419 Fangxie Road, Shanghai, 200011 China

**Keywords:** Epidural labor analgesia, Labor progress, Maternal outcomes, Neonatal outcomes

## Abstract

**Background:**

Whether epidural administered sufentanil combined with low-concentration ropivacaine affected labor progress as well as maternal and neonatal outcomes still remained unknown. The aim of this study was to assess the impact of epidural sufentanil plus ropivacaine on maternal and neonatal outcomes.

**Methods:**

This is a retrospective cohort study. Data of singleton full-term pregnancy women who received epidural labor analgesia for vaginal delivery from May 2018 to June 2020 were collected. Parturients were divided into two groups (the R group and the SR group) according to different medication regimens for epidural labor analgesia. The implementation of epidural analgesia during labor was performed with 0.167 % ropivacaine in the R group and 0.1 % ropivacaine in combination with 0.5 µg/ml sufentanil in the SR group. The primary outcome of our study included the duration of labor progress and the incidence of maternal fever, postpartum hemorrhage, fetal distress and neonatal Apgar scores less than 7 at 1 and 5 min. The secondary outcome included the incidence of episiotomy, instrumental delivery, caesarean section and grade III meconium-stained amniotic fluid.

**Results:**

There were a total 3778 deliveries during the study period, 1994 and 1784 parturients were included in the R group and in the SR group, respectively. The length of the first stage of labor was remarkably shorter in the R group in comparison to the SR group (548.0 ± 273.0 vs. 570.9 ± 273.0, *P* = 0.013). No significant difference was found in the incidence of maternal fever, postpartum hemorrhage, fetal distress and in the neonatal Apgar scores less than 7 at 1 and 5 min between two groups. Other Maternal outcomes were comparable in the R group and the SR group.

**Conclusions:**

0.5 µg/ml sufentanil plus 0.1 % ropivacaine for epidural labor analgesia prolonged the duration of the first stage of labor, but did not have additional impact on maternal and neonatal outcomes compared with the sole 0.167 % ropivacaine.

**Trial registration:**

Clinical Research Information Service with registration number ChiCTR2100045162. Registered 7 April 2021.

## Background

Epidural analgesia with local anesthetics such as ropivacaine was considered as an effective way to relieve the pain of uterine contraction [1–3]. However, some studies [4, 5] have shown that a relatively high total dosage of local anesthetic may have an effect on uterine activity by decreasing the rate and strength of the uterine contraction.

Moreover, local anesthetics were more likely associated with epidural-related maternal fever via a number of ways of immunomodulation [6, 7] and cell injury [8]. Thus, epidural administered local anesthetic might potentially cause complications on both maternal and neonatal outcomes time-dependently and dose-dependently. Epidural opioids was also recommended and commonly used in combination with local anesthetics to reduce the dose of local anesthetics and to provide equal, if not superior analgesic effect [9]. Wang et al. reported that ropivacaine with sufentanil for labor analgesia provided effective analgesic effect without significant side effects and delay of the labor progress [10]. But in a recent meta-analysis [11], sufentanil in combination with bupivacaine was considered to be associated with worse neonates Apgar score.

In our institution, as a regular protocol, epidural labor analgesia is conducted using either 0.167 % ropivacaine or 0.1 % ropivacaine in addition to 0.5 µg/ml sufentanil. Although both of them provided almost the equivalent analgesic effects, whether the combination epidural sufentanil and low-concentration ropivacaine will affect the labor progress as well as maternal and neonatal outcomes, as the single usage of high-dose local anesthetic, still remained controversial.

Therefore, we performed a retrospective cohort study to observe women who received 0.167 % ropivacaine alone vs. sufentanil with 0.1 % ropivacaine for epidural labor analgesia to assess the impact of the combination of sufentanil and ropivacaine on both maternal and neonatal outcomes.

### Methods

This retrospective group study was approved by the institutional review board from the Obstetrics and Gynecology Hospital of Fudan University and was registered at the Clinical Research Information Service (ChiCTR2100045162, Principal investigator: Yue Li). Informed consent of information collection for research was obtained from all participants. All the study methods were carried out in accordance with the combination of our institutional regulations and guildlines. The study adheres to the ethical standards of the Declaration of Helsinki (1964) and its subsequent amendments.

Medical records were retrospectively reviewed from May 2018 to June 2020, singleton full-term pregnancy women who received epidural labor analgesia via vaginal delivery were included in the study. Women with severe systemic disease that results in functional impairment (American Standards Association, ASA ≥ III) or fetal dysplasia were excluded from the cohort. Parturients were classified into two groups: women who received 0.167 % ropivacaine alone (the R group) and women who received sufentanil with 0.1 % ropivacaine (the SR group) according to different medication regimens for epidural labor analgesia by provider preference. Both regimens were administered via an epidural analgesia pump in a patient-controlled analgesia manner, 8 ml/h epidural analgesic with 0.167 % ropivacaine or 0.1 % ropivacaine in combination with 0.5ug/ml were administered in the R group or the SR group, respectively. The patient-controlled bolus was set to deliver a dosage of 8 ml of analgesics with a lockout interval of 15 min.

Demographic characteristics were collected, including the maternal age, gestational weeks, parity in the gravidae, body mass index (BMI), whether the mother underwent induced labor, presence of pregnancy comorbidities, and neonatal birth weight.

The primary outcome of the present study was the duration of labor progress. The duration of labor progress collected in our study included three stages. The first stage of labor was defined as time from diagnosis of labor to full dilatation of the cervix. The second stage of labor was defined as time from full dilatation of cervix to delivery of the fetus and the third stage of labor was time from delivery of the fetus to delivery of the placenta. The secondary outcome included the incidence of maternal fever, postpartum hemorrhage, fetal distress, episiotomy, instrumental delivery, caesarean section, grade III meconium-stained amniotic fluid (MSAF) and neonatal outcome (Apgar scores < 7, at 1 and 5 min, respectively). Maternal fever was defined as the body temperature > 38℃. Postpartum hemorrhage was defined as estimated blood loss ≥ 500 ml after delivery of the placenta. Fetal distress was diagnosed by the obstetricians and their Apgar scores were assessed by neonatologists after vaginal deliveries and cesarean deliveries. All these demographic information and obstetric outcomes were collected from the electronic medical record system of our hospital.

### Statistical analysis

Statistical analysis was performed using SPSS 20.0 (*SPSS, Inc., Chicago, IL, USA*) and GraphPad Prism 5.01 software (*GraphPad Inc., CA, USA*). The duration of three stages of labor were presented as mean ± standard deviation (SD). Neonatal outcomes, incidences of maternal fever, postpartum hemorrhage, instrumental delivery, conversion to caesarean section, postpartum hemorrhage the use of episiotomy and the presence of grade III MSAF were presented as percentage. Continuous variables were analyzed using t test, and categorical variables were analyzed using Chi-Square test. Statistical significance was set at *P* < 0.05.

## Results

According to the inclusion criteria, a total of 3778 deliveries during the study period were retrospectively reviewed, with 1994 parturients in the R group and 1784 parturients in the SR group. The demographic characteristics including the maternal age, BMI, gestational weeks, rate of multiparous and complications, oxytocin induction and neonatal birth weight did not demonstrate significant differences between the R group and the SR group (*P* > 0.05) (Table 1).

**Table 1:** Demographic characteristics of study population. Data reported as mean ± SD and N (%)

	R (*n* = 1994)	SR (*n* = 1784)	*P*
Maternal age (y)	29.77 ± 3.93	30.04 ± 3.99	0.103
BMI	26.85 ± 3.287	26.59 ± 3.176	0.117
Gestational weeks	39.29 ± 1.051	39.33 ± 1.086	0.158
Multiparous	233 (13.2)	252 (12.5)	0.507
Complications	488 (27.7)	533 (26.4)	0.385
Oxytocin induction	752 (37.7)	698 (39.1)	0.373
Birth weight(g)	3377 ± 394.9	3381 ± 407.5	0.605

*P*-value is compared between the R group and the SR group

*y *Year, *BMI *Body mass index, *g *Gram

As shown in Table 2, the duration of the first stage of labor was significantly shorter in the R group in comparison to the SR group (548.0 ± 273.0 vs. 570.9 ± 273.0, *P*=0.013). No significant difference was detected in the duration of the second stage of labor and the third stage of labor between the two groups (*P*> 0.05). The incidence of maternal fever, postpartum hemorrhage, episiotomy, instrumental delivery, conversion to caesarean section, and grade III MSAF also had no statistical significance between the two groups (*P*> 0.05).

**Table 2:** Labor progress and maternal outcomes. Data reported as mean ± SD and N (%)

	R (*n* = 1994)	SR (*n* = 1784)	*P*
Labor progress			
1st stage (min)	548.0 ± 273.0	570.9 ± 273.0	0.013*
2nd stage (min)	68.51 ± 50.36	66.61 ± 55.25	0.082
3rd stage (min)	5.36 ± 3.16	5.29 ± 3.34	0.112
Maternal fever	451 (22.62)	412 (23.09)	0.728
Postpartum hemorrhage	78 (3.91)	74 (4.15)	0.712
Episiotomy	896 (51.41)	755 (48.27)	0.072
Instrumental delivery	193 (11.07)	187 (11.96)	0.426
Caesarean section	251 (12.5)	220 (12.3)	0.812
Grade III MSAF	417 (20.91)	377 (21.13)	0.869

*min* Minute, *MSAF *Meconium-stained amniotic fluid

*Statistical significance of difference between the R group and the SR group (*P*<0.05)

For neonatal outcomes (Table 3), the incidence of fetal distress demonstrated no statistical significance between the R group and the SR group. There was no significant difference for the Apgar score between the two groups at 1 and 5 min as well.

**Table 3:** Adverse neonatal outcomes. Data reported as N (%)

	R (*n* = 1994)	SR (*n* = 1784)	*P*
Fetal distress	231 (11.58)	228 (12.78)	0.262
Apgar score			
1 min Apgar score < 7	34 (1.71)	33 (1.85)	0.737
5 min Apgar score < 7	3 (0.15)	3 (0.17)	0.891

## Discussion

Epidural administered ropivacaine and sufentanil has been a common protocol used in labor analgesia [12, 13]. Adding sufentanil to ropivacaine can effectively enhance the analgesia effect [14] and thus reduce the consumption of the local anesthetics. However there are concerns of the opioids-related adverse effects on the labor progress as well as maternal and neonatal outcomes [15].

In our study, the average duration of the first stage of labor was significantly lower in the 0.167 % ropivacaine group than the 5 µg sufentanil + 0.1 % ropivacaine group. This was similar in several clinical studies [16, 17] which found that women with opioids analgesia had a prolonged duration of labor. Some animal studies also showed that opioids can affect spontaneous contraction of uterine musclature [18, 19]. It seems to be possible that the delay of labor progress could be partly attributed from the inhibiting effect of opioids on uterine contractility. However, a study about the effects of opioids on isolated human pregnant uterine muscles demonstrated that fentanyl and morphine inhibited spontaneous contraction of the uterine muscle, while sufentanil did not [20]. The mechanism of the inhibitory potency of opioids was not yet clear. It was believed that the inhibitory effect of opioids was not mediated through the opioid receptors. Since the dosage of the opioids used on the isolated human uterine muscles was far more than the regular clinical doses, it is somewhat unclear that the clinical doses of opioids can be the culprit prolongs the labor progress with clinical significance or increases the risk of postpartum hemorrhage by decreasing the uterine contractility. In accordance to our study, although the first stage of labor was longer in the sufentanil combination group, the statistical significance between the two groups was not clinically important (9 h and 8 min vs. 9 h and 30 min) and the incidence of postpartum hemorrhage was shown to have no difference between the two groups. Moreover, there was no difference in the incidence of maternal fever, postpartum hemorrhage, episiotomy, instrumental delivery, caesarean section and grade III MSAF. Regarding the maternal outcomes, the combination of 0.1 % ropivacaine and 5 µg/ml sufentanil for epidural labor analgesia was not able to bring advantages or disadvantages in comparison to 0.167 % ropivacaine.

Other than that, neonatal outcomes were also demonstrated no significant difference between the two groups, which wasn’t fully compliant to the study with Wang et al. [21]. In their study, it was found that the sole local anesthetic ropivacaine had less incidence of poor Apgar scoring at 1 min than the combination of ropivacaine and sufentanil, which could be resulted from the low concentration of ropivacaine in the SR group in our study. In some previously published prospective studies comparing the impact of opioids in labor analgesia, the concentration of the local anesthetics in the intervention group and the control group were usually the same, in order to isolate and clearly identify the effect of the opioids administration. But in clinical scenarios, we found that the addition of sufentanil was able to reduce the dosage of ropivacaine almost by half, which provided comparable analgesic effect. Therefore, in the present study, the concentration of ropivacaine in the SR group was lower than the R group. What we actually retrospectively reviewed and compared was two different therapeutic regimens in management of labor pain. Similar to the findings from several studies [22–24], our results showed that 0.5 µg/ml sufentanil plus 0.1 % ropivacaine had no influence on neonatal outcomes.

There were several limitations in our study. First, the dosage of oxytocin, the incidence of fetal hypoxia and admission to the neonatal intensive care unit (NICU) and maternal adverse effects such as vomiting and pruitus could not be collected due to limited to available data. Secondly, the medication regimens for epidural labor analgesia were commonly used in our single institution, which might not be popularized entirely to other institutions with various labor analgesia managements. And it is difficult to come to a conclusion that any differences we found in our study is simply resulted from the addition of sufentanil. Lastly, the data of this retrospective study were collected from the electronic medical record, which made it somewhat challenging to confirm the exactitude.

## Conclusions

In conclusion, in this retrospective study, an epidural infusion of 0.1 % ropivacaine + 0.5 µg/ml sufentanil was associated with a longer duration of 1st stage of labor than an epidural infusion of 0.167 % ropivacaine, but did not have additional impact on either maternal or neonatal outcomes.

## Data Availability

The datasets generated and analyzed during the current study are available from the corresponding author on reasonable request.

## Abbreviations

ASA: American Standards Association
BMI: Body mass index
MSAF: Meconium-stained amniotic fluid
SD: Standard deviation
NICU: Neonatal intensive care unit
